# Unveiling Face Recognition Challenges and Awareness in Autism Spectrum Disorder: Insights from the Italian Famous Face Test (IT-FFT)

**DOI:** 10.1007/s10803-025-06879-9

**Published:** 2025-05-16

**Authors:** Martina Ventura, Valerio Manippa, Alessandro Oronzo Caffò, Giovanni Cicinelli, Emanuela Nobile, Roberto Keller, Davide Rivolta

**Affiliations:** 1 The MARCS Institute for Brain, Behaviour, and Development, Western Sydney University, Sydney, Australia; 2 Department of Education, Psychology and Communication, University of Bari Aldo Moro, Bari, Italy; 3 Adult Autism Center, Department of Mental Health, Local Health Unit ASL Città di Torino, Torino, Italy

**Keywords:** Prosopagnosia, Developmental disorders, Face recognition, Metacognition, Social cognition, Autism

## Abstract

Accurate face recognition is crucial for navigating social interactions. While neurotypical individuals generally show no issues with face processing, persons with Autism Spectrum Disorder (ASD) often exhibit impairments in this area. This study explores the extent of these face recognition deficits in autistic adults, focusing on their ability to identify famous faces, along with the awareness (metacognition) of their face recognition skills. Using the Italian Famous Face Test (IT-FFT) and the Prosopagnosia Index-20 (PI-20), to compare face recognition performance and self-awareness of face recognition abilities between 50 non-autistic and 49 individuals diagnosed with level 1 ASD. Autistic people had significantly lower face identification scores and greater difficulties recognizing famous faces than non-autistic participants. Additionally, autistic individuals reported more face recognition challenges on the PI-20, highlighting their awareness of these deficits. These findings suggest that face recognition impairments in ASD extend to famous faces and underscore the importance of further research to explore targeted interventions aimed at improving different aspects of face recognition in autistic people.

The human face represents the stimulus we rely on the most for social interactions, as it provides rich social information such as identity, emotions, and intentions (Haxby et al., 2000). Thus, accurate and rapid face processing and recognition are necessary for building and maintaining social relationships, as they allow one to modulate behavior, and intent, and provide an appropriate social response (Avery et al., 2016). Indeed, it has been shown that superior social skills (e.g., extraversion personality trait) are linked to better face identification (Li et al., 2010). In contrast, deficiencies in face processing can lead to anxiety and social situation avoidance (Yardley et al., 2008). Difficulty with face recognition is a characteristic associated with various conditions, including Autism Spectrum Disorder (ASD). ASD is a developmental disorder characterized by compromised communication and basic social development, along with narrowed interests and stereotyped, repetitive behaviors (American Psychiatric Association & Association, 2013).

Numerous studies have consistently highlighted abnormalities in face processing among autistic individuals, manifesting with a wide range of severity challenges, from mild to moderate impairments (Teunisse & de Gelder, 2003; Zhao et al., 2016), differently affecting face memory, face perception, or facial expression recognition (Stantić et al., 2022; Yeung, 2022; Lievore et al., 2025). This and other social deficits significantly impact autistic people’s ability to navigate human interactions (Constantino, 2011) - but see Tracy et al., 2011 and Naumann et al., 2018 for a different perspective. Despite these variations, a recent meta-analysis by Griffin et al., 2021 consistently points towards deficits in face recognition skills among autistic individuals. These difficulties could occur for several reasons. For instance, a visual bias toward local or featural information might interfere with the configural processing necessary for face recognition (Robertson & Baron-Cohen, 2017). Additionally, reduced social motivation combined with behaviors such as eye avoidance limits visual engagement with faces, exacerbating these challenges (Tanaka & Sung, 2016; Kamensek et al., 2024). Notably, face recognition impairment in ASD shares similarities with prosopagnosia, a condition characterized by the inability to explicitly recognize familiar people’s identities based on their faces (Manippa et al., 2023; Rivolta et al., 2013). Recent research suggests that up to one-third of autistic individuals show prosopagnosia-like symptoms (Cygan et al., 2018). However, there seem to be distinct etiologies for face recognition challenges within ASD compared to prosopagnosia. Indeed, while some cases may resemble prosopagnosia, others may involve broader social cognition deficits or underlying mechanisms (Kamensek et al., 2023).

However, in everyday life, we view faces in various contexts where the recognition of familiar or unfamiliar faces is required. That implies that we deal with different kinds of familiarity; for instance, differentiating between a family member and a stranger or a TV star. Research indicates that our ability to recognize faces varies significantly depending on their familiarity (Matthews et al., 2018). Herzmann et al. (2004) found that reaction times were quicker for both famous and personally familiar faces compared to unknown faces, but only the personally familiar triggered a strong skin conductance response. Additionally, the neurological basis for recognizing different types of faces varies significantly, with several studies showing distinct brain activation patterns or modulation for unknown, famous, and personally familiar faces (Andrews et al., 2017; Caharel et al., 2014; Gosling & Eimer, 2011; Keyes et al., 2010). This suggests the need to distinguish between experimentally learned/unknown, famous, and personally familiar faces when assessing face perception, as some conditions may show difficulties in some, but not all, aspects of face processing (Liccione et al., 2014). However, ASD studies on face recognition usually employed experimental familiarization techniques (Ventura et al., 2024) with initially unfamiliar face stimuli (e.g., Cambridge Face Memory test - CFMT, (Duchaine & Nakayama, 2006), mostly using Old-new paradigms, and only a few studies used famous or personally familiar identities (Weigelt et al., 2012). Thus, even though it’s not a formal diagnosis criterion, improving research on how autistic people (and more broadly, people with recognition impairment) process different types of faces could offer a unique perspective on comprehending the mechanisms behind their different social activity and inform the development of targeted interventions.

Another relevant aspect to consider is metacognition, which plays a critical role in people’s performance and behaviors across several domains and tasks, including visual cognition (Rhodes, 2019; Rahnev, 2022). Metacognition is broadly defined as the ability to be aware and in control of one’s mental processes, skills, and performance (Metcalfe and performance (Metcal Shimamura, 1994), which is important for effective decision-making and strategy implementation, directly affecting performance and outcomes in different cognitive activities (Amran et al., 2021). Metacognition studies on autistic individuals demonstrate overall metacognitive difficulty in various cognitive domains, such as memory (Teunisse & de Gelder, 2003; Zhao et al., 2016). However, as ASD represents a heterogeneous condition for the etiology, phenotype, and outcome (Masi et al., 2017), not all autistic individuals may experience the same type of metacognitive difficulties (Embon et al., 2023). Specifically, visual metacognition is the ability to evaluate one’s performance on visual perceptual tasks, such as recognizing our ability to distinguish between colors or shapes accurately. What is so far fairly unexplored is whether autistic people have insights into their face recognition skills, with only one study (Gehdu et al., 2024) showing a statistically significant correlation between the Prosopagnosia Index-20 scores of autistic individuals and their performance on two variants of the CFMT. Assessing this aspect is important at the theoretical level since it informs cognitive theories of ASD, but also at the practical level since working on metacognition could represent an intervention tool in the domain of face recognition, as studies suggest that metacognitive skills can be trained (Fleur et al., 2021; Taouki et al., 2022).

Thus, our study aims to (i) ascertain whether individuals diagnosed with ASD level 1 according to DSM 5 criteria (APA, 2013) show famous face recognition deficits through the recently developed Italian Famous Face Test (IT-FFT, (Ventura et al., 2024); and (ii) whether autistic people have insights into their overall face identification skills. We hypothesized that autistic people would show worse performance in famous face recognition, as well as decreased metacognition awareness compared to non-autistic people.

Additionally, we also evaluate the potential of using the IT-FFT as a tool to highlight face recognition deficits, further enhancing our diagnostic capabilities and intervention strategies.

## Methods

### Participants

A total of 99 adult participants (ages 18–57 years, *M* = 29.41, *SD* = 10.98) were included in the study, divided into 49 clinical and 50 control participants. Their years of schooling ranged from 7 to 21 years (*M* = 14.20, *SD* = 2.70), and all had normal or corrected-to-normal vision.

The clinical group consisted of 49 individuals diagnosed with ASD level 1 recruited from the Regional Center for Adults with Autism in the Piedmont region (Italy). Each of them was diagnosed with level 1 ASD by psychologists and psychiatrists of the Center according to DSM-5 criteria (American Psychiatric Association & Association, 2013) based on clinical anamnesis, clinical interview, psychiatric interview, psychopathology assessment, cognitive assessment with the Wechsler Adult Intelligence Scale (WAIS-IV, (Wechsler, 2012) or Leiter-3 (Roid & Miller, 1997), diagnostic evaluation with the Autism Diagnostic Interview (ADI-R, Rutter et al., 2003), and Autism Diagnostic Observation Schedule - Second Edition (ADOS-2, Lord et al., 2000) or Autism Asperger Diagnostic Scale (Ritvo et al., 2011; Keller et al., 2020). The control sample consisted of 50 non-autistic individuals recruited from local student communities by snowball sampling and stratified to match the ASD group’s age, sex, and education level (see Table 1). To ensure that no individual selected for the control sample fell within the autism spectrum, they completed the Italian version of the Autism-Spectrum Quotient (AQ; (Baron-Cohen et al., 2001; Ruta et al., 2012). All NTs scored between 12 and 26, which is below the recommended cut-off of 29, indicative of clinically significant autism traits (*M* = 16.09, *SD* = 4.40).

**Table 1:** The two groups showed no significant differences in sex distribution (assessed using a chi-squared test), age, or years of schooling (both assessed using independent samples *t*-tests). The last rows report the mean and standard deviation scores for intelligence quotient (assessed using the Wechsler adult intelligence scale or Leiter-3), autism diagnostic Interview-Revised (ADI-R), autism diagnostic observation Schedule–Second edition (ADOS-2), or the Ritvo autism asperger diagnostic scale (RAADS) for the autistic sample. The *N* reported next to each value indicates the number of autistic individuals diagnosed using that specific instrument. For the non-autistic sample, the autism-Spectrum quotient score is reported

	Autistic (*N* = 49)	Non-autistic (*N* = 50)	*p*-value
Sex (N)	22 F, 27 M	26 F, 24 M	0.480 (Χ^2=^ 0.500)
Age (M ± SD)	29.47 ± 10.12	29.36 ± 11.86	0.961 (t = 0.049)
Schooling yr (M ± SD)	13.77 ± 2.27	14.62 ± 3.04	0.121 (t=-1.156)
Intelligence Quotient (M ± SD)	102.06 ± 18.27		
ADI-R_Social Inter (M ± SD)	12.26 ± 6.75 (*N* = 19)		
ADI-R_Comm and Lang (M ± SD)	7.31 ± 5.45 (*N* = 19)		
ADI-R_Restr/Rep Behav (M ± SD)	4.26 ± 3.26 (*N* = 19)		
ADI-R_Abnorm of Develop < 36 months (M ± SD)	1.37 ± 1.86 (*N* = 19)		
ADOS (M ± SD)	11.80 ± 5.89 (*N* = 5)		
RAADS (M ± SD)	127.16 ± 29.98 (*N* = 44)		
Autism-Spectrum Quotient (M ± SD)		16.90 ± 4.40	

Individuals with a history of neurological diseases, cerebral stroke, epilepsy or epileptic seizures, head injury with loss of consciousness, severe medical conditions or psychiatric disorders (this latter criterion was not applied to ASD group, as they often present comorbidity), and alcohol or drug abuse were excluded.

### Procedure and Measurement

The study was approved by the Ethical Committee of the University of Bari (protocol number: ET-19-01) and was performed following the Helsinki Declaration and its later amendments. After providing informed consent and socio-demographic information, each participant took the Italian version of the Prosopagnosia Index-20 (PI-20) (Tagliente et al., 2023), a self-report assessment of face recognition ability. The PI-20 consists of twenty statements that represent facial recognition experiences (e.g., “I have always had a bad memory for faces”; “I feel like I frequently offend people by not recognizing who they are”). Respondents rate the accuracy of these statements on a five-point scale (1 being “Totally disagree” and 5 being “Totally agree”). Scores range from 20 to 100, with higher scores indicating greater difficulties in facial recognition. The PI-20 internal consistency in the present study was assessed using Cronbach’s alpha, yielding a value of 0.88.

After, each participant completed the Italian Famous Face Test (IT-FFT; Ventural et al., 2024), administered through a computer-based interface. The IT-FFT comprises 100 images: 50 depicting celebrities and 50 unknown people. Faces were presented one at a time in randomized order at the center of the screen. Participants were instructed to view each photograph carefully and (i) evaluate whether the face was famous or not (*Face familiarity*), and (ii) provide the name of the celebrity if they thought the face was famous or specific information about the character (*Face identification*). At the end, the final checklist containing all the 50 names belonging to the famous people included in the IT-FFT was shown, and participants had to indicate the names of celebrities they were unfamiliar with (i.e., people they do not know and, thus, they cannot recognize) (*Name familiarity*). A detailed description of the stimuli, procedures, index measures, and normative data can be found in Ventura et al. (2024). The IT-FFT internal consistency in the present study was assessed using Cronbach’s alpha, yielding a value of 0.96.

*Face Identification* score was computed by summing the number of correctly identified celebrities, which could be achieved either by naming the celebrity or providing specific biographical information, such as referring to them as “the main character of the film…”. Minor name spelling errors were not counted as mistakes. However, responses that were missing, incorrect, or too general (e.g., simply stating “actor” or “singer”) were given a score of 0. The final accuracy for face identification was expressed as a percentage, calculated by dividing the face identification score by the name familiarity score (assessed through the checklist) and then multiplying by 100. This method ensured that participants’ accuracy reflected their performance relative to their self-reported knowledge of famous personalities, rather than a fixed number of targets. The *Face Familiarity* scores, instead, were based on signal detection theory, which categorizes outcomes into four types: false alarms (incorrectly identifying non-famous faces as famous), correct rejections (correctly identifying non-famous faces as non-famous), misses (failing to recognize famous faces), and hits (correctly recognizing famous faces). The sensitivity measure, d-prime (*d’*), was calculated from these outcomes, with higher values indicating greater sensitivity to recognizing famous faces. Additionally, response bias (*β*) was computed to understand participants’ tendencies: a *β* of 1.00 indicates no bias, values below 1.00 indicate a liberal tendency (more likely to report faces as famous), and values above 1.00 indicate a conservative tendency (more likely to report faces as non-famous).

### Data Analysis

Our dependent variables were PI-20 scores and the IF-FFT *Face familiarity* and *Face identification* scores. To determine whether autistic samples exhibit famous face recognition deficits and whether they have insight into their overall face identification skills, we conducted 4 generalized linear models, one for each dependent variable (Face identification, PI-20 score, Face familiarity (*d’*), and response bias (β) scores). For each dependent variable, we run an Analysis of Covariance (ANCOVA) with groups (autistic vs. non-autistic) and sex (F vs. M) as between factors, and age and schooling as covariates. Additionally, we computed a 2 × 2 Chi-squared analysis to assess the distribution of autistic and non-autistic samples above (ES > 1) and below (ES = 0) the FFT-IT *Face Identification* cutoff scores (as defined by Ventura et al., 2024). Finally, Pearson’s correlation between Face Identification and PI-20 was calculated for each sample to evaluate whether autistic and non-autistic samples have insights into their overall face identification skills.

## Results

**Fig. 1:**
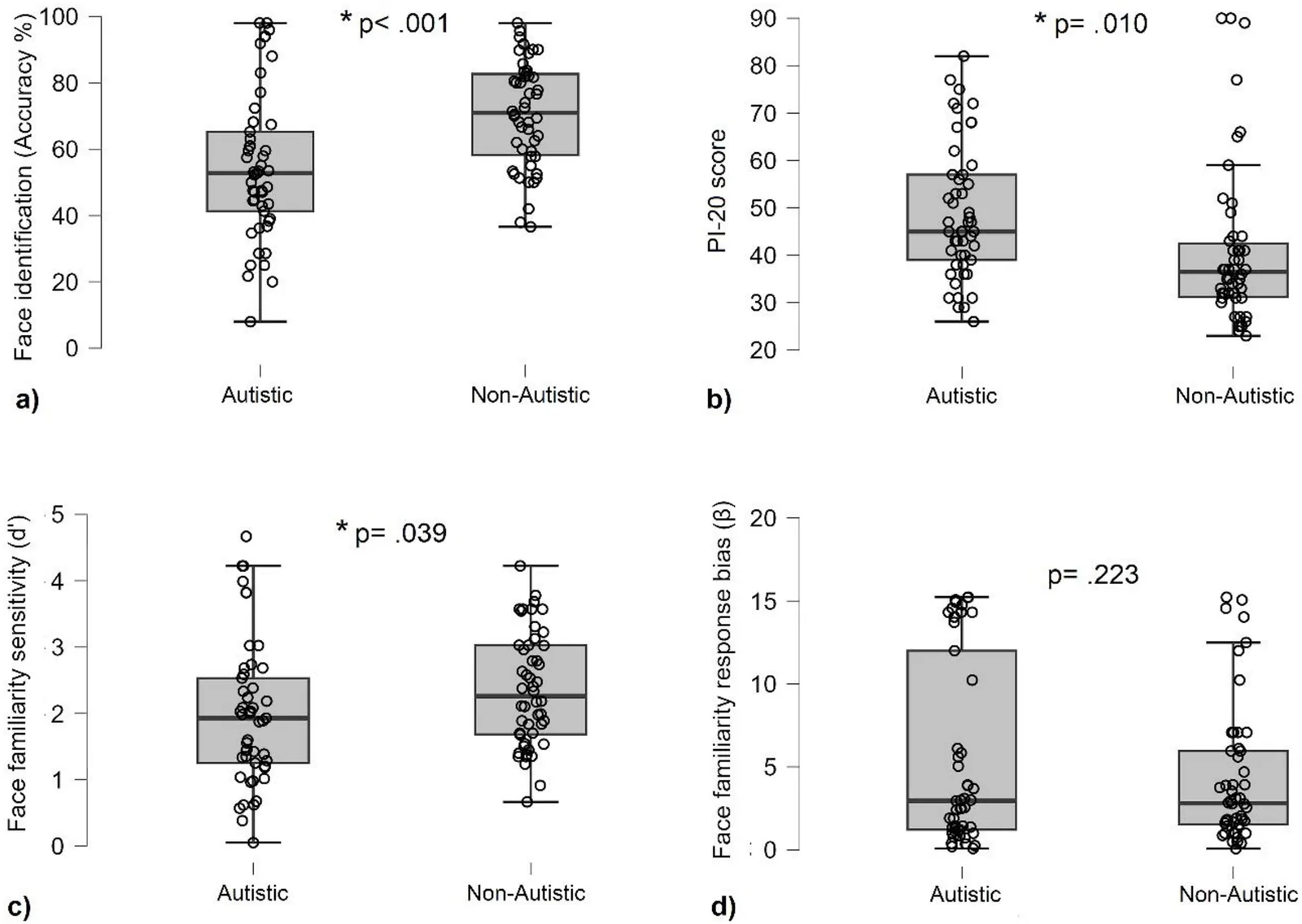
The boxplots show the comparisons between autistic and non-autistic individuals, derived from the 4 ANCOVAs conducted on: (**a**) Face identification accuracy (%)—higher in non-autistic compared with autistic participants, (**b**) Prosopagnosia Index-20 (PI-20) scores—higher in autistic than non-autistic participants, (**c**) Face familiarity sensitivity (d’)—significantly lower in the ASD group compared to the non-autistic group, and (**d**) Face familiarity response bias (β)—showing no group difference. Each boxplot displays the distribution of individual data points (i.e., the dots), the median (i.e., the central line within the box), and the 25th -75th interquartile range (the box). The whiskers (error bars) extend to the minimum and maximum data points within 1.5 times the interquartile range

The ANCOVA on *Face identification* scores showed a main effect of the group (F_1,94_= 19.049, *p* <.001, *η*_*p*_^2^ =.167, see Fig. 1a) with a higher face identification score for non-autistic compared with autistic group, and a significant effect of participants’ age (F_1,94_= 10.790, *p* =.001, *η*_*p*_^2^ =.103), indicating that face identification increased along with participants’ age. The ANCOVA conducted on PI-20 score showed a main effect of the group (see Fig. 1b) (F_1,94_= 6.854, *p* =.010, *η*_*p*_^2^ =.078), with autistic scoring significantly higher on PI-20 than non-autistic participants. The ANCOVA conducted on Face familiarity (*d’*), showed a main effect of group (F_1,94_= 4.352, *p* =.039, *η*_*p*_^2^ = 0.062) with higher *d’* score for non-autistic compared with autistic participants (see Fig. 1c), and a main effect of participants’ age (F_1,94_= 11.598, *p* <.001, *η*_*p*_^2^ = 0.099) with increasing *d’* scores along with participants’ age. The ANCOVA on face familiarity response bias (*β*) showed no effects (see Fig. 1d).

All the ANCOVAs results are reported in Table 2.

**Table 2:** Results of ANCOVAs (F-value, p-value, partial Eta squared) for face identification (accuracy), PI-20 scores, sensitivity (*d*′), and response bias (*β*)

ANCOVAs	Face identification			PI-20			Sensitivity (d’)			Response bias (β)		
Factors	F	*p*	$\eta^2_p$	F	*p*	$\eta^2_p$	F	*p*	$\eta^2_p$	F	*p*	η²_*p*_
Group	19.049	< 0.001	0.167	6.845	0.010	0.078	4.352	0.039	0.062	1.502	0.223	0.014
Sex	0.266	0.607	< 0.001	0.001	0.996	0.001	1.679	0.199	0.012	0.223	0.638	0.003
Age	10.790	0.001	0.103	0.276	0.600	0.005	9.687	0.001	0.099	1.655	0.201	0.018
Schooling yr	2.559	0.113	0.028	2.296	0.090	0.029	1.645	0.209	0.023	0.019	0.892	< 0.001

**Fig. 2:**
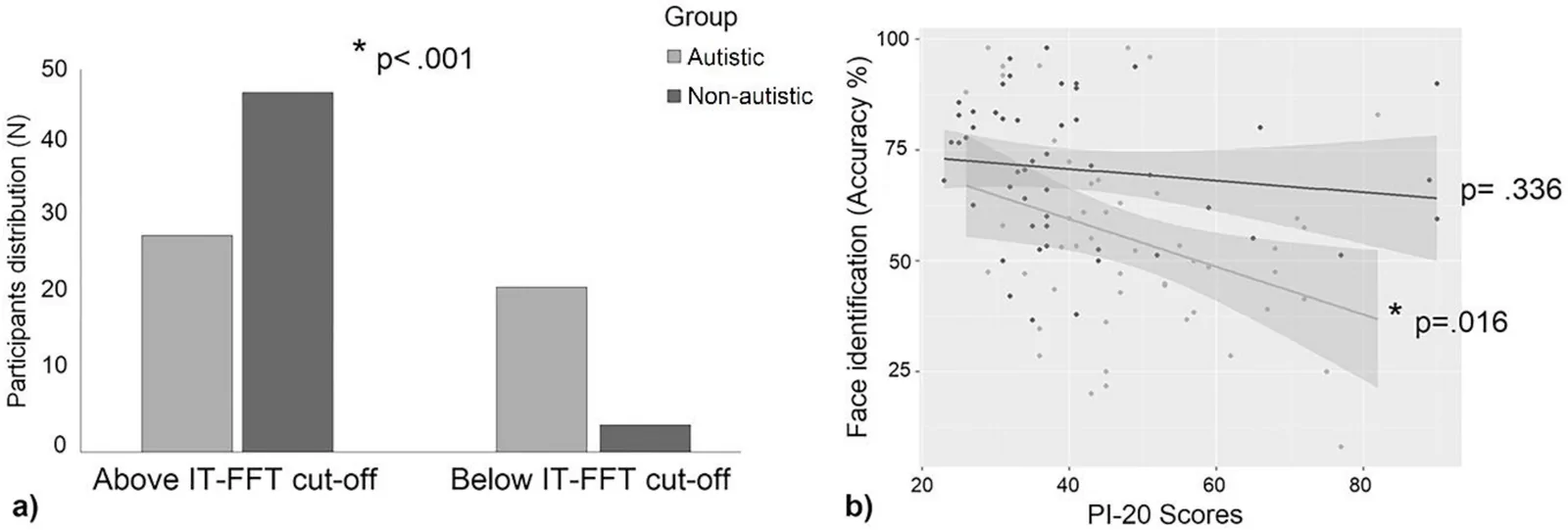
(**a**) Distribution of autistic participants (in light gray) and non-autistic participants (in dark light gray) above and below the IT-FFT Face Identification cut-off: a significantly higher number of ASD individuals score below the cut-off compared to the NTs; (**b**) Correlation between Face Identification and PI-20 scores: the negative correlation is significant within the autistic sample but not within the non-autistic sample

The 2 × 2 chi-squared tests conducted to assess the distribution of autistic and non-autistic participants above and below the IT-FFT Face Identification cut-off, demonstrated a higher proportion of autistic distributed below the cut-off score (*N* = 21, 42.8%) compared to the non-autistic participants (*N* = 3.6%) (χ^2^_1_ = 18.305; *p*<. 001) (see Fig. 2a). Descriptive data and comparisons are reported in Table 2. Finally, a significant negative correlation was found between Face identification and PI-20 scores (*r*= -.314, *p* =.002, see Fig. 2b), driven primarily by the autistic people sample (*r* = -.343, *p* =.016). No significant correlation was observed in the non-autistic group (*r* = -.139, *p* =.336).

## Discussion

In this study, we investigated potential differences in face recognition skills between individuals diagnosed with ASD level 1 and non-autistic individuals using the IT-FFT. We also assessed their level of insight into their own face recognition ability through the PI-20. Our findings showed that autistic performed significantly worse than non-autistic people in the IT-FFT and reported more subjective face recognition difficulties to the PI-20. Furthermore, PI-20 and IT-FFT accuracy scores correlated (negatively) in autistic but not in non-autistic individuals. This pattern highlights a connection between perceived abilities and objective performance in face identification: while the latter seems strongly impaired, the former appears to be unaffected by the presence of ASD.

The significant main effect showing higher face identification accuracy in the non-autistic group suggests that autistic individuals struggle more with recognizing famous faces, consistent with previous research on facial recognition difficulties in ASD (Tanaka et al., 2012; Weigelt et al., 2012). Notably, more than 40% of autistic participants (compared to just 4% of the non-autistic sample) scored below the IT-FFT cut-off (Ventura et al., 2024), indicating pronounced and significant difficulties in facial recognition skills. Additionally, the non-autistic sample achieved higher *d’* scores compared to the ASD sample, reinforcing the idea that autistic people demonstrate reduced discriminative abilities when judging face familiarity. It is important to note that response bias, as measured by *ꞵ* score, was comparable between autistic and non-autistic individuals, suggesting that the difference in d’ scores is not due to differences in response tendencies. These findings emphasize the need to consider a weakened sense of familiarity as a contributing factor to face processing impairments due to ASD. While both face identification accuracy and face familiarity sensitivity were influenced by participant sex, according to the normative data of the IT-FFT (Ventura et al., 2024), the absence of significant interaction effects suggests that factors such as sex, age, and education did not significantly modulate the observed group differences.

These results show the heterogeneous nature of face recognition challenges within the autistic population, where some people may experience more severe impairments than others. Difficulties in face recognition not only hinder social interactions and communication but also disrupt the formation of social bonds, which are essential for navigating everyday life (Davis, 2010; Lane et al., 2018). Specifically, impairments in face recognition can affect an individual’s ability to form meaningful connections (Davis, 2010; Lane et al., 2018), maintain relationships, and fully participate in everyday activities as people struggling with face recognition impairments often report chronic anxiety about offending others. This anxiety, combined with feelings of embarrassment, guilt, and failure, can further hinder social functioning and lead to increased stress and isolation (Yardley et al., 2008; Barton, 2003). Given that social skills are already compromised in ASD, it’s essential to further explore how these face recognition difficulties manifest in real-world scenarios and develop tailored assessments and targeted face recognition training programs to effectively address these issues and enhance the social functioning of autistic people.

Moreover, we assessed the recognition ability insight of autistic individuals, which has rarely been investigated. Metacognition is a broad concept that includes several components (Jia et al., 2019); among these, one important aspect is the individuals’ thoughts, beliefs, and reflections on one’s cognitive capacity, which could also be defined as metacognitive monitoring (Carpenter & Williams, 2023). Metacognition studies in ASD led to mixed results; some research reports no differences between non-autistic and autistic participants’ metacognition levels (Nicholson et al., 2021; Williams et al., 2018), while others suggest that they show several difficulties in metacognitive abilities (Maras et al., 2020; Sawyer et al., 2014). However, it is unclear whether metacognition is domain-general or domain-specific (Faivre et al., 2018; Rouault et al., 2018). Considering metacognition to be domain-specific, it may be possible that this ability in autistic people may be disrupted for some domains or tasks and not for others, and this could explain the variability in results observed among autistic individuals.

To this aim, we administered the Italian version of the PI-20. Contrary to our expectation, our results showed that autistic had higher PI-20 scores compared with non-autistic participants, indicating that autistic individuals report more difficulties but at the same time are aware of their struggles with face recognition. This result aligns with previous literature that showed no impaired metacognition among autistic people (Adams et al., 2020; Ambrus et al., 2019; Migo et al., 2012) and specifically, Gehdu et al. (2024), which observed significant correlations between PI-20 scores and autistic individuals performance on two variants of the CMFT.

Additionally, a moderate negative correlation between PI-20 scores and face recognition accuracy was observed when considering all participants together. However, this correlation was significant only within autistic and not within the non-autistic sample. This suggests that autistic individuals who perceive themselves as having difficulties in recognizing faces tend to perform worse in face recognition tasks, meaning that their self-awareness reflects the actual performance. This higher metacognitive ability observed in autistic participants can be partly attributed to the lack of extreme scores reported by the non-autistic sample. In contrast, the greater variability in face recognition abilities characterizing ASD (mostly impaired), allows for a more pronounced correlation between self-reported difficulties in face recognition and actual performance. Accordingly, the correlation between PI-20 scores and face recognition performance seems primarily driven by those with either very low (e.g., autistic participants) or very high face recognition abilities (Bindemann et al., 2014; Estudillo & Wong, 2021; Palermo et al., 2017). Thus, while the PI-20 can serve as a useful tool for initial screening and assessing individuals’ insight into their face recognition skills, its discriminative power for identifying prosopagnosia-level performance on objective assessments may be limited. This highlights the need to balance caution against overreliance on self-report measures with recognizing their utility in specific contexts. Integrating subjective and objective assessments is crucial for comprehensively understanding face recognition difficulties (Arizpe et al., 2019).

## Conclusions, Limitations, and Future Research

This study found that individuals diagnosed with ASD level 1 performed worse than matched non-autistic people to the IT-FFT, coherently with the PI-20 assessing self-reported face recognition difficulties. IT-FFT and PI-20 negatively correlated mainly in the autistic sample, demonstrating that they are aware of their face recognition abilities. Therefore, metacognitive skills related to facial recognition appear to be preserved in autistic people; however, this finding is specific to facial recognition and does not encompass other aspects of metacognition. Future studies should consider exploring additional metacognitive abilities, using IQ as a covariate, as higher support levels are often associated with intellectual disabilities, making broader comparisons challenging. Furthermore, while this test can provide insights into the ability to recognize well-known faces and assess identification skills and familiarity, our data analysis strategy may only partially capture the comprehensive aspects of face recognition abilities, as other tasks related to face recognition were not assessed. Our test and others such as face-matching tests or the CFMT (Duchaine et al., 2006; Duchaine & Nakayama, 2006) can provide a more comprehensive insight into individual face identification dimensions (i.e., face perception, face memory, and face recognition).

In conclusion, this study highlights the IT-FFT as a valuable tool for detecting face recognition deficits in ASD and potentially other conditions, such as neurodegenerative disorders (e.g., Alzheimer’s disease and frontotemporal dementia), or prosopagnosia, which often present challenges in facial recognition (Manippa et al., 2023). While preliminary results are encouraging, further research is needed to evaluate the IT-FFT’s effectiveness across a broader range of conditions to enhance our diagnostic capabilities and understanding. Moreover, additional work should not only focus on autistic people’s face recognition and metacognition but also extend to a broader range of cognitive abilities, including memory, attention, executive functioning, problem-solving skills and general visual cognition, providing a more comprehensive understanding of their cognitive profiles and how these abilities relate to face recognition deficits.
